# Extrusion Processing of Fungal-Contaminated Cereals as a Method for Spore Reduction and Binder Development in Feed Materials

**DOI:** 10.3390/ma18225117

**Published:** 2025-11-11

**Authors:** Paweł Cwalina, Sławomir Obidziński, Małgorzata Kowczyk-Sadowy, Aneta Sienkiewicz, Jacek Mazur

**Affiliations:** 1 Department of Agri-Food Engineering and Environmental Management, Bialystok University of Technology, Wiejska 45E, 15-351 Bialystok, Poland; s.obidzinski@pb.edu.pl (S.O.); m.kowczyk@pb.edu.pl (M.K.-S.); a.sienkiewicz@pb.edu.pl (A.S.); 2 Department of Food Engineering and Machines, University of Life Sciences in Lublin, Głębocka 28, 20-612 Lublin, Poland; jacek.mazur@up.lublin.pl

**Keywords:** extrusion, sustainable feed production, feed pellets, feed processing, microbiological safety

## Abstract

The increasing demand for safe and durable feed materials highlights the need for processing methods that simultaneously enhance physical quality and reduce microbiological contamination. Extrusion technology offers a promising solution by combining thermal and mechanical effects that improve binding performance while inactivating fungal spores present in cereal grains. In this study, maize, barley, sorghum, soybean, and wheat grains naturally contaminated with fungal spores were subjected to extrusion prior to pelleting. The physical properties of the resulting pellets, including bulk density, physical density, and kinetic durability, were evaluated and compared with those obtained from ground (non-extruded) grains. Pellets containing extruded grains generally exhibited higher physical density, with the highest value recorded for pellets containing extruded mould-infected sorghum grain (1179.82 kg·m^−3^) and the lowest for pellets containing healthy soybeans (1063.63 kg·m^−3^). The kinetic durability of extruded cereal pellets increased on average by 4.02%, enhancing their resistance to mechanical stress during transport and storage. Microbiological analyses confirmed a significant reduction in fungal colony-forming units (CFUs) after extrusion and pelleting, ranging from 27% to 65%, depending on the cereal type. The most pronounced reduction was observed in maize-based pellets contaminated with mould spores, decreasing from 1.70 × 10^5^ to 6.03 × 10^4^ CFU·g^−1^. These results demonstrate that extrusion is an effective method for producing cereal-based feed materials with improved physical quality and enhanced microbiological safety, contributing to more sustainable feed production.

## 1. Introduction

Cereal grains are a fundamental raw material in feed production. However, due to their susceptibility to filamentous fungi, they are frequently exposed to microbiological contamination and the accumulation of mycotoxins. This contamination limits their further use in animal nutrition and increases the risk of economic losses [1,2]. Ten-year monitoring data have shown that in a significant proportion of feed and feed material samples—such as maize, wheat, and soybeans—the concentration of aflatoxin B_1_ (AFB_1_) exceeded the permissible limit of 20 µg·kg^−1^. Exceedances were reported in 41.1% of samples from South Asia, 38.5% from Sub-Saharan Africa, and 20.9% from Southeast Asia [3].

A particular challenge involves managing low-quality grain batches in which the presence of fungal spores often precludes their direct use in feed manufacturing processes. One promising method for processing such materials is extrusion, which combines high temperature, pressure, and shear forces. This process not only modifies the physical and functional properties of cereals but also reduces the number of viable microorganisms and, under certain conditions, may partially degrade selected mycotoxins [4,5].

In the context of feed production, extrusion enables the effective utilisation of contaminated grain batches by improving the physical properties of the resulting feed pellets and increasing their microbiological safety. This approach aligns with the principles of sustainable agriculture and the circular economy. Extrusion is a modern processing technology that, due to its high efficiency and versatility, allows for the modification of physicochemical and functional properties of agricultural raw materials [6,7,8]. It is defined as a continuous unit operation in which material is subjected to elevated temperature, pressure, and shear forces before being forced through a die [9,10]. In feed manufacturing, extrusion improves pellet parameters such as density and mechanical durability while simultaneously reducing the number of fungal spores, thereby enhancing the microbiological safety of the final product. In addition, the process contributes to reduced water and energy consumption, further supporting sustainable feed production [11]. This technology allows the use of cereal grains contaminated with fungal spores and other low-quality raw materials for the production of nutritionally improved feeds [12,13,14].

One alternative use for mouldy cereals is their utilisation as a substrate in agricultural biogas plants. In Germany, approximately 9600 biogas plants are currently in operation, most of which use plant-based raw materials such as maize silage and grasses. Due to its high gas yield, maize accounts for around 74% of the substrate mix [15]. Mould-contaminated cereals can serve as an alternative or supplementary substrate, particularly in large installations that often source feedstocks externally. Although the presence of mycotoxins (including deoxynivalenol and zearalenone) can affect fermentation performance, it does not entirely exclude the use of such materials, provided that appropriate substrate ratios are maintained and biological process parameters are controlled. This approach helps reduce raw material losses while supporting renewable energy production [16,17,18].

Another potential route for managing mouldy cereals is thermal processing through combustion or co-combustion in energy installations. Research has shown that fungal growth causes substantial changes in the material structure, including an increase in particle porosity and specific surface area (from approximately 18.8 m^2^·g^−1^ in healthy grain to 256.4 m^2^·g^−1^ in heavily mould-infested grain). These changes directly affect ignition properties and combustion behaviour. The larger reaction surface enhances oxidation, accelerates smouldering and combustion, and increases the risk of self-ignition [19]. Mouldy grain can be effectively used as a biomass fuel, provided that appropriate flue gas cleaning and emission control technologies are applied. This solution enables the utilisation of waste material, reduces raw material losses, and supports renewable energy generation.

While most previous works focused primarily on mycotoxin degradation efficiency, the present research investigates extrusion as a pre-conditioning step enabling the safe utilization of fungal-spore-contaminated grains in compound feed production. The study combines both microbiological (spore reduction) and technological (pellet quality and binding properties) aspects, demonstrating extrusion as a dual-function process—improving hygienic safety and enhancing pellet durability. This addition clarifies the original contribution and differentiates our work from earlier studies on extrusion–mycotoxin interactions. The aim of this study was to evaluate the feasibility and effects of extrusion as a pre-processing method for cereal grains contaminated with fungal spores (maize, barley, sorghum, soybean, and wheat) used in the production of feed pellets. The study focused on assessing the impact of extrusion on both the physical properties and microbiological safety of the pellets, in response to the growing demand for durable and hygienically safe feed materials. A specific objective was to determine the effect of extrusion on density, kinetic durability, and fungal contamination levels of pellets containing 15% of mould-infested cereal grains.

## 2. Materials and Methods

### 2.1. Materials

**Figure 1:**
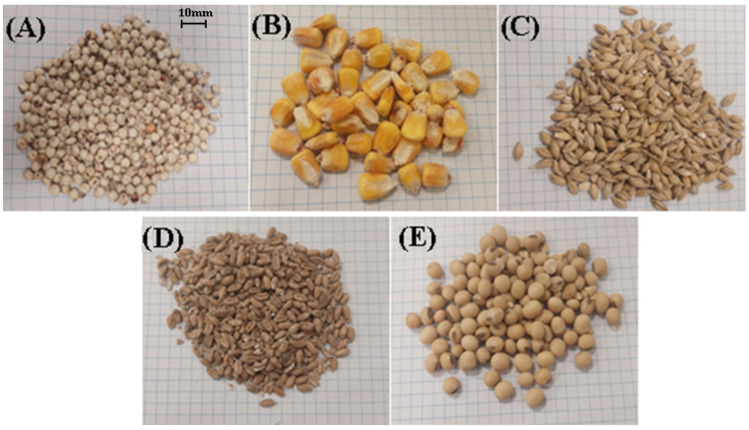
Raw materials (own photos): (**A**) sorghum (Dari), (**B**) maize (Aldo), (**C**) barley (Julia), (**D**) wheat (Euforia), (**E**) soybean (Adessa).

Experimental feed mixtures were prepared using commonly available plant-based ingredients and cereal grains naturally contaminated with fungal spores. The basal mixture consisted of meadow hay, wheat bran, and multi-fruit pomace (peels, pulp, seeds, and stems of apples and pears). Cereal grains (sorghum, soybean, maize, barley, and wheat) contaminated with fungal spores were added to each mixture (Figure 1).

All ingredients were purchased from a local supplier (Białystok, Poland) and stored under controlled, dry conditions prior to processing. Fungal-contaminated grains were obtained from naturally infected batches during storage and were visually classified based on the presence of fungal growth on the grain surface.

### 2.2. Moisture Content Determination

The moisture content of the raw materials was determined following the procedure specified in the PN-EN ISO 18134-1:2015-11 standard [20]. Measurements were conducted using an AXIS AGS moisture analyser. For each material, five independent determinations were performed at a controlled temperature of 105 °C to ensure reliable and representative results. The mean value of these measurements was calculated and reported as the final moisture content.

### 2.3. Extrusion of Contaminated Cereal

**Figure 2:**
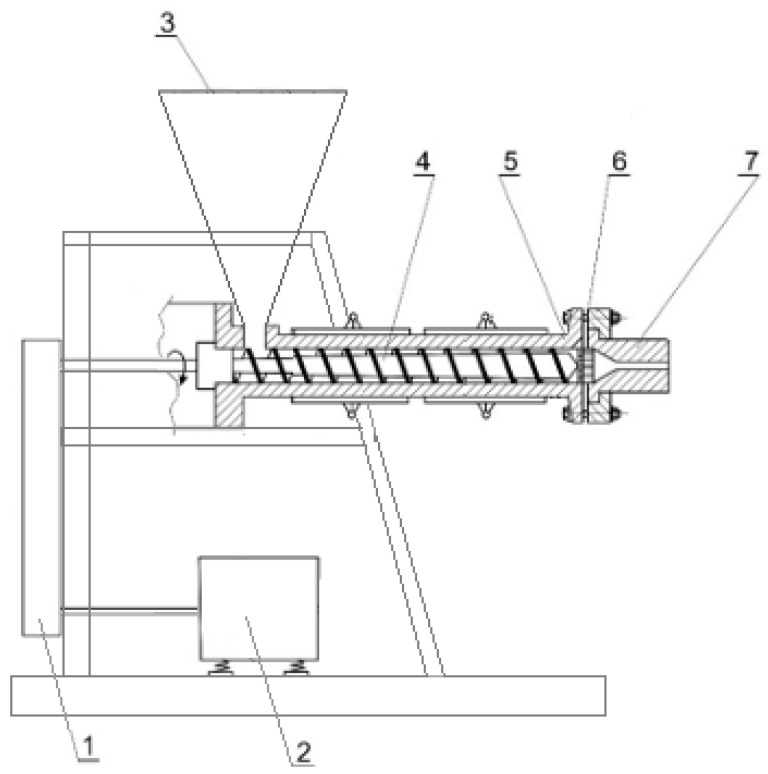
E-100 extruder station diagram (own elaboration based on [22]): 1—belt transmission, 2—motor, 3—feed hopper, 4—screw feeding the material to the head, 5—extruder body, 6—head mounting assembly, 7—head body.

The extrusion process was carried out using a single-screw extruder E-100 (Presoil, Poland) (Figure 2). During extrusion, the mould-contaminated cereal grains were subjected to high pressure (20–30 atm) and elevated temperature (110–140 °C), resulting in intensive mechanical breakdown of the grains and the instantaneous evaporation of water upon exiting the die [21].

The constant parameters during the experiment were as follows:

- *w_m_* = 21—mixture moisture content [%],
- *d*_0_ = 6—head diameter [mm],
- *Q_m_* = 20—mixture mass flow rate [kg·h^−1^],
- *T* = 140—temperature in the extrusion chamber [°C],
- *p* = 20–40—compaction pressure during pellet formation [atm],
- screw configuration: single screw,
- screw speed: 200 rpm,
- residence time: 25 s.

### 2.4. Pressure Agglomeration Process

The pressure agglomeration (pelletising) of feed mixtures containing cereal grains contaminated with fungal spores (Table 1) was carried out using the SS-5 test stand. A detailed description of the apparatus and its operating principles is provided in a previous publication [23].

During the pelletising experiments, all process parameters were kept constant to ensure repeatability and enable a reliable assessment of the physical properties of the resulting pellets:

- *w_m_* = 19—mixture moisture content [%],
- *d*_0_ = 6—diameter of the matrix holes [mm],
- *d_m_* = 216—matrix diameter [mm],
- *h_m_* = 32—matrix height [mm],
- *Q_m_* = 100—mixture mass flow rate [kg·h^−1^],
- *n_r_* = 270—rotational speed of the densifying roller system [rpm],
- *h_r_* = 0.4—gap between the rollers and the matrix [mm].

### 2.5. Measurement of Physical and Bulk Density of Pellets

The physical density of the pellets was evaluated using a representative set of ten randomly selected samples. Prior to measurement, the pellet edges were gently leveled with an EINHELL WSG-125E angle grinder (Landau, Germany) to obtain uniform shapes and ensure dimensional accuracy. The length and diameter of each pellet were then measured using a caliper with an accuracy of 0.05 mm. Subsequently, the individual pellet mass was determined on an analytical balance with a precision of ±0.001 g. Based on these data, the physical density (*ρ_g_*) of each pellet was calculated using Equation (1), relating its mass to the measured geometrical dimensions:

(1) $$\rho_{g}=\frac{m_{g}}{V_{g}}$$

where

*ρ_g_*—physical density of pellets [kg∙m^−3^],
*m_g_*—mass of pellets [kg],
*V_g_*—volume of tested pellets [m^3^].

The volume of pellets was calculated using Equation (2):

(2) $$V_{g}=\pi \cdot r^{2}\cdot h\;\left[m^{3}\right]$$

where

*r*—radius of pellets [m],
*h*—height of pellets [m].

The bulk density of the pellets was determined following the PN-EN ISO 17828:2016-02 standard [24]. A container of known volume was filled with the test material until completely occupied, ensuring minimal voids. The mass of the filled container was then recorded using an analytical balance with an accuracy of ±0.1 mg. Bulk density was calculated by dividing the mass of the pellets by the internal volume of the container.

### 2.6. Determination of the Kinetic Strength of the Pellets

The mechanical durability of the pellets was determined in accordance with the PN-EN ISO 17831-1:2016-02 standard [25]. A Holmen NHP100 tester (Norfolk, UK) was used for the measurements. Before testing, the pellet samples were passed through a 5 mm sieve to remove any loose fines. A 100 g portion of the cleaned material was then placed into the testing chamber, where the pellets were exposed to a stream of air that generated repeated impacts both between the pellets and against the perforated chamber walls. After completing the test cycle, the material was re-sieved, and the mass of the remaining intact pellets was recorded. The kinetic durability index was calculated using Equation (3):

(3) $$P_{dx}=\frac{m_{2}}{m_{1}}\cdot 100\;$$

where

*P_dx_*—kinetic strength of pellets [%],
*m*_1_—sample weight before test [kg],
*m*_2_—sample weight after test [kg].

The constant values during the research were:

- *T* = 60—duration of the test [s],
- *p* = 7—target pressure [kPa].

### 2.7. Determination of Mycotoxins by LC–MS/MS

The quantitative analysis of mycotoxins in the examined feed mixtures was carried out using liquid chromatography coupled with tandem mass spectrometry (LC–MS/MS). Representative samples were collected after extrusion and pelletisation, then finely ground and homogenised to ensure sample uniformity. The measurements were performed using a 6420 LC/MS Triple Quadrupole system (Agilent Technologies, Santa Clara, CA, USA), ensuring high sensitivity and selectivity for the targeted mycotoxins.

Chromatographic separation was performed on a 1260 Infinity LC system (Agilent Technologies) using a Poroshell 120 EC-C18 column (3.0 × 100 mm, 2.7 µm) (Santa Clara, CA, USA). The injection volume was 20 µL. The mobile phase consisted of (A) 95% water with 0.025% TFA and 5% acetonitrile, and (B) 95% acetonitrile with 0.025% TFA. A linear gradient was applied from 90% A at 0 min to 90% B at 15 min, with a constant flow rate of 0.5 mL·min^−1^.

The LC–MS/MS analysis was conducted using an ESI source operating at 350 °C, with a drying gas flow of 9.5 L·min^−1^, nebulizer pressure of 45 psi, and capillary voltage of 4000 V. The instrument operated in MRM mode, with two transitions (quantifier and qualifier) monitored per analyte. Quantification was based on matrix-matched external calibration, and method performance was verified through LOD/LOQ determination, recovery experiments, and analysis of fortified quality control samples.

### 2.8. Determination of Microbial Contamination

In a portion of the tested cereal and crop grain samples, no macroscopic signs of mould contamination were observed. The grains exhibited a normal appearance, colour, and surface structure, indicating the absence of external fungal infection symptoms. In contrast, the remaining samples were contaminated with mould fungi; the grains were clumped together, discoloured, covered with a characteristic fungal growth, and emitted a musty odour. A microbiological analysis of all samples was performed to determine the actual level of fungal contamination.

Samples weighing 10 g were collected in accordance with the PN-EN ISO 24333:2012 standard [26]. The enumeration of mould colonies was carried out using the decimal dilution method, which involved preparing a series of sample dilutions followed by surface plating on Martin medium supplemented with Bengal rose. This medium supports the selective growth of moulds and yeasts while inhibiting bacterial development. All samples were plated in triplicate, and incubation was carried out at 26 ± 1 °C for 5 days.

After incubation, the grown fungal colonies were counted, and the results were expressed as colony-forming units per gram of sample (CFU·g^−1^).

### 2.9. Statistical Analysis

Most of the results are expressed as means ± standard deviation (SD) from triplicate measurements. Hierarchical cluster analysis was performed to construct a dendrogram that grouped the data into a cluster tree based on the distances between all pairs of objects. The clustering was carried out using Euclidean distance, with Ward’s method applied as the agglomeration criterion. In addition, simultaneous clustering of both objects and features was conducted, and the results were visualized as a colour similarity map. All statistical analyses were performed using Statistica 13.3 software (TIBCO Software Inc., Palo Alto, CA, USA).

## 3. Results

### 3.1. Moisture Content

Based on the measurements, the moisture content of the raw materials used in the study ranged from 9.35% to 12.76% (Table 2). Among the unprocessed materials, maize exhibited the highest moisture level (12.76 ± 0.18%), followed by meadow hay (12.51 ± 0.19%) and sorghum (12.09 ± 0.21%). The lowest moisture content was recorded for fruit pomace (9.35 ± 0.26%), which may affect the flow properties and compaction behaviour of the mixture during the pelletising process.

Cereal grains infected with fungal spores, after undergoing extrusion, exhibited significantly lower moisture levels compared to their raw counterparts. The moisture content of extruded wheat, barley, maize, soybean, and sorghum decreased to 8.82 ± 0.08%, 8.16 ± 0.12%, 9.82 ± 0.08%, 9.46 ± 0.10%, and 9.28 ± 0.12%, respectively. Moisture reduction occurred mainly as a result of rapid depressurization and flash evaporation at the die exit.

According to technical recommendations for pellet production [27], raw materials with moisture contents below approximately 12% tend to exhibit reduced plasticity, which may hinder proper agglomeration and lead to process instability. Preliminary tests performed prior to pelleting indicated that meadow hay-based feed mixtures require a higher moisture content—around 20%—to ensure adequate compaction and stable pellet formation. Therefore, additional water was added to the mixtures before pelleting to provide sufficient binding properties and maintain stable operation of the pelleting unit.

### 3.2. Effect of Extruded Fungal-Spore-Infected Grains on the Pelleting Process and Pellet Properties

Table 3 presents the results of the analysis of feed mixtures containing cereal grains infected with fungal spores after extrusion. The study assessed the effect of incorporating these extruded materials on key parameters of the pelleting process, including the power consumption of the pelletiser during compaction, as well as the physical properties of the resulting pellets, namely mechanical durability, bulk density, and physical density.

Based on the obtained results (Table 3), it was found that the incorporation of extruded cereal grains contaminated with fungal spores into feed mixtures significantly affected both the pelleting process and the physical properties of the resulting pellets. A clear influence on the power demand of the pelletiser was observed. Depending on the type of grain, the pelletiser power demand during compaction ranged from 2.45 to 2.83 kW. The lowest power demand was recorded during pelleting of mixtures containing extruded soybean (2.45 kW), while the highest value was noted for mixtures with extruded maize (2.83 kW). These differences can be attributed to variations in the composition and structural properties of the extruded raw materials, which affect their compressibility during densification. Previous studies have indicated that optimising feedstock properties and reducing the proportion of fines can lower the energy demand during pelleting [28,29,30].

Çitil et al. [31] investigated the power requirements, capacity, and selected physical properties of pellets produced using a flat-die pelletising machine with an inlet diameter of 8 mm, an outlet diameter of 5 mm, and a die diameter of 195 mm. Two feedstocks—barley and a compound feed mixture—were pelleted to evaluate performance differences. The pelleting capacity reached 172.80 kg·h^−1^ for barley and 170 kg·h^−1^ for the mixed feed. The required electrical power was 7.63 kW during barley pelleting and increased to 9.18 kW for the compound feed, indicating a higher energy demand associated with more complex formulations. The specific energy consumption was 0.044 kW·kg^−1^ for barley and 0.054 kW·kg^−1^ for the mixed feed, demonstrating the influence of raw material properties on the energy intensity of the pelleting process.

Although extrusion is generally more energy-intensive than pelleting, it provides simultaneous thermal–mechanical treatment and microbial decontamination, thereby reducing the need for additional processing steps or chemical treatments. Based on typical industrial data, the specific energy consumption (SEC) of cereal extrusion ranges from 0.08 to 0.15 kWh·kg^−1^, compared with 0.02 to 0.05 kWh·kg^−1^ for pelleting [12]. While extrusion involves higher energy input, this can be offset by the elimination of separate grain drying operations, as the process effectively handles materials with higher initial moisture. Moreover, extrusion enables the safe utilization of substandard or fungal-contaminated grains that would otherwise be discarded, thereby reducing feed losses and improving overall resource efficiency. From a life-cycle perspective, these combined benefits can compensate for the additional energy cost and contribute positively to the environmental footprint of feed production.

Currently, there are no dedicated ISO or PN standards for feed pellets. However, several international standards originally developed for solid biofuels are commonly used in feed pellet research to determine bulk density, mechanical durability, and moisture content [20,24,25]. Additionally, industry guidelines such as those from FEFAC and national feed regulations specify quality requirements including minimum mechanical durability (>90%), moisture content (10–14%), and particle size uniformity [32].

The results (Table 3) confirmed a significant effect of incorporating extruded cereal grains on the physical properties of pellets compared with those produced using ground grains. Pellets containing extruded cereal grains (B variants) generally exhibited higher physical density than those containing ground grains (A variants). The highest physical density was observed for pellets containing extruded sorghum (4B), reaching 1179.82 kg·m^−3^, while the lowest value was recorded for pellets with ground soybean (5A), at 1063.63 kg·m^−3^. Similar density ranges were reported by Peng et al. [33], who found that typical feed pellets exhibit a physical density between 1000 and 1250 kg·m^−3^ depending on moisture content, particle size distribution, and formulation.

The bulk density of the produced feed pellets ranged from 397.47 to 412.58 kg·m^−3^ (Table 3). The lowest bulk density was observed for pellets containing 15% mould-infected maize (3B) at 397.47 kg·m^−3^, whereas the highest value was recorded for pellets containing 15% wheat (2A) at 412.58 kg·m^−3^. However, the differences between the bulk densities of pellets obtained from the various mixtures were relatively small, not exceeding 15 kg·m^−3^. This indicates that the applied pelleting parameters ensured a relatively uniform packing structure of the produced pellets.

Compared with typical bulk density values reported for feed pellets—usually in the range of 500 to 700 kg·m^−3^ depending on composition and processing parameters [34,35,36]—the obtained values were considerably lower. This may be attributed to the specific composition of the tested mixtures, which were based mainly on meadow hay. This component likely affected particle shape and surface properties. Lower bulk density may also result from a lower degree of compaction during pelleting, associated with the fibrous structure of meadow hay, which contributes to higher porosity.

A comparison of the kinetic durability index (PDIH) values of pellets produced from mixtures containing healthy grains (A variants) and mould-infected grains (B variants) revealed clear differences (Table 3). For all analysed formulations, the PDIH values were higher in the B variants than in their corresponding A variants. The highest PDIH value was recorded for pellets containing 15% mould-infected wheat (1B), reaching 82.33%, which represents a 3.22% increase compared with the control (1A, 79.11%). Similar trends were observed for the other variants: 2B—75.26% (an increase of 1.75% compared with 2A), 3B—74.78% (7.54% increase), 4B—65.65% (2.50% increase), and 5B—79.82% (5.08% increase).

The increase in PDIH across all B variants can be attributed to the extrusion process applied to the contaminated grains. Extrusion, through the combined effects of high temperature and pressure, induces starch gelatinisation and partial protein denaturation, thereby enhancing their binding properties and improving pellet cohesion [37,38]. This effect was particularly evident in variant 3, where the difference between A and B was the largest (7.54%).

Massuquetto et al. [39] studied the effect of starch gelatinisation on the quality of maize and soybean meal-based poultry feed pellets. The authors demonstrated a clear relationship between conditioning time and PDIH values. The lowest PDIH (90.41%) was recorded in non-conditioned samples, while steam conditioning for 60 and 80 s increased PDIH to 95.78% and 95.43%, respectively. Prolonging the time to 100 and 120 s did not result in further improvement, and the values remained stable at 95.16% and 95.07%. These findings clearly indicate that an appropriate degree of starch gelatinisation, achieved through optimal conditioning, significantly improves pellet mechanical durability.

Abadi et al. [40] investigated the effect of fat type and level added in the mixer, as well as different pellet binders, on the physical quality of poultry feed pellets. The highest PDIH (84.6%) was obtained for diets containing 1.5% CFP (calcium fat powder), whereas the lowest (56.9%) was recorded for diets with 3% SO (soybean oil). On average, CFP-containing mixtures exhibited higher PDIH values (76.8%) than those with SO (62.9%). Increasing the fat content from 1.5% to 3% reduced PDI by more than 13 percentage points.

Lower PDIH values obtained for some variants may be linked to the high proportion of meadow hay in the feed mixture. Due to its fibrous structure, this raw material produces a wider particle size distribution after grinding, making it more difficult to achieve sufficient inter-particle bonding during pellet formation. Long, irregular fibres may act as “micro-wedges”, weakening the pellet structure and increasing susceptibility to mechanical damage during handling and transport. Previous research has clearly shown that finer grinding of feed components leads to pellets with higher PDIH, primarily due to improved surface contact and bonding between particles [41,42,43]. Consequently, the proportion of coarse fibrous raw materials such as meadow hay may be a limiting factor in achieving high physical pellet quality.

### 3.3. Mycotoxin Reduction Efficiency of the Extrusion–Pelletisation Process

The analysis of mycotoxin content in the feed pellets revealed clear differences between the variants prepared with healthy cereal grains (A) and those containing 15% mould-infected grains (B). In all cases, significantly higher mycotoxin concentrations were recorded in the B variants (Table 4).

In this study, no mycotoxin determinations were performed on the raw cereal grains, as their levels have already been extensively characterised in numerous previous research works on contaminated cereal raw materials. Existing literature clearly indicates that mould-infected grain can represent a significant source of mycotoxins, such as aflatoxin B_1_ (AFB_1_), deoxynivalenol (DON), zearalenone (ZEN), fumonisin B_1_ (FB_1_), and ochratoxin A (OTA) [44,45,46,47]. For this reason, the focus was placed on the practical aspect of the technological process—assessing the effect of adding contaminated grains to the final feed mixture on the resulting mycotoxin content after thermo-mechanical processing. This approach allows for a direct evaluation of contamination effects under realistic feed production conditions and a better assessment of potential risks to animal health.

For the 1A–1B pair, a clear increase in all analysed mycotoxins was observed after adding mould-infected grain. DON concentrations increased from 118.12 µg·kg^−1^ to 315.83 µg·kg^−1^, FB_1_ from 11.37 µg·kg^−1^ to 68.11 µg·kg^−1^, AFB_1_ more than tripled (from 0.83 µg·kg^−1^ to 2.64 µg·kg^−1^), and OTA rose from 3.18 µg·kg^−1^ to 42.19 µg·kg^−1^. A particularly large increase was recorded for trichothecenes: T-2 toxin increased from 17.43 µg·kg^−1^ to 99.10 µg·kg^−1^, and HT-2 toxin from 7.42 µg·kg^−1^ to 37.00 µg·kg^−1^. ZEN content increased more than eightfold, from 2.13 µg·kg^−1^ to 17.30 µg·kg^−1^. All values remained below the EU regulatory limits (DON: 5000 µg·kg^−1^, fumonisins: 20,000 µg·kg^−1^, AFB_1_: 20 µg·kg^−1^), while OTA (42.19 µg·kg^−1^) approached the recommended threshold level of 50 µg·kg^−1^ [48].

For the 2A–2B pair, the differences were even more pronounced. DON increased from 209.58 µg·kg^−1^ to 418.23 µg·kg^−1^, FB_1_ from 12.45 µg·kg^−1^ to 258.11 µg·kg^−1^, and fumonisin B_2_ (FB_2_) from 16.82 µg·kg^−1^ to 309.65 µg·kg^−1^. AFB_1_ and OTA reached for 2B 3.97 µg·kg^−1^ and 37.74 µg·kg^−1^, respectively. T-2 increased from 14.32 µg·kg^−1^ to 183.00 µg·kg^−1^, HT-2 from 2.63 µg·kg^−1^ to 29.73 µg·kg^−1^, and ZEN from 3.62 µg·kg^−1^ to 18.95 µg·kg^−1^. Although these values remained below regulatory limits, they indicate a potential risk of subclinical effects on animal health [49].

In samples 3A–3B, DON increased from 189.63 µg·kg^−1^ to 538.37 µg·kg^−1^, while FB_1_ and FB_2_ increased from 9.38 µg·kg^−1^ and 12.63 µg·kg^−1^ to 112.65 µg·kg^−1^ and 283.19 µg·kg^−1^, respectively. T-2 and HT-2 increased from 18.93 µg·kg^−1^ and 4.63 µg·kg^−1^ to 153.00 µg·kg^−1^ and 38.17 µg·kg^−1^. AFB_1_ levels quadrupled, reaching 4.13 µg·kg^−1^, while OTA rose from 4.98 µg·kg^−1^ to 41.52 µg·kg^−1^. These levels remained below the regulatory limits but confirmed the significant impact of contaminated grain on final mycotoxin content [50].

The highest DON level was recorded in the 4A–4B pair, increasing from 318.90 µg·kg^−1^ to 842.48 µg·kg^−1^. Fumonisins rose from 7.52 µg·kg^−1^ and 9.85 µg·kg^−1^ to 187.52 µg·kg^−1^ and 198.82 µg·kg^−1^, respectively. T-2 increased from 11.86 µg·kg^−1^ to 152.10 µg·kg^−1^, and HT-2 from 3.52 µg·kg^−1^ to 61.50 µg·kg^−1^. OTA for 4B reached 32.98 µg·kg^−1^, while AFB_1_ reached 4.82 µg·kg^−1^. Although mycotoxin levels did not exceed legal limits, they were clearly higher than typical background levels found in healthy grains, indicating substantial contamination of the raw material.

For the 5A–5B pair, DON increased from 239.11 µg·kg^−1^ to 430.38 µg·kg^−1^. Fumonisins reached the highest levels of all samples (FB_1_: 265.12 µg·kg^−1^; FB_2_: 205.83 µg·kg^−1^). T-2 and HT-2 reached 231.00 µg·kg^−1^ and 67.10 µg·kg^−1^, approximately four times higher than the control variant. AFB_1_ and OTA increased to 4.20 µg·kg^−1^ and 38.17 µg·kg^−1^, respectively.

Hajnal et al. [51] investigated the influence of extrusion process parameters on the reduction of multiple mycotoxins in triticale grain. All tested extrusion settings resulted in measurable mycotoxin reduction. DON reduction ranged from 0.12% to 16.6%, while 3-AcDON, 15-AcDON, and HT-2 were reduced by 1.7–32.8%, 1.7–45.7%, and 24.3–60.5%, respectively. Alternariol monomethyl ether (AME) showed the highest reduction rates (53.2–91.8%), with maximum values achieved at a screw speed (SS) of 500 rpm, feed rate (FR) of 26 kg·h^−1^, and moisture content (MC) of 20 g/100 g. For 3-AcDON and 15-AcDON, the highest reductions (32.8% and 45.7%) were obtained at the maximum screw speed (800 rpm) combined with low or high feed rates and variable moisture content, while HT-2 reduction peaked at 60.5% at medium screw speed and low moisture content. These results indicate that extrusion can significantly reduce mycotoxin concentrations in cereal products when optimised for specific compounds, providing an effective means of limiting contamination while maintaining acceptable product quality.

Khan et al. [52] examined the physical properties of pellets after extrusion (true density, water stability, sinking velocity), which are important for rumen escape in ruminants. They showed that process control (e.g., screw speed, end cooling) allows for the production of pellets with high density and stability, optimising starch digestion site and nutrient utilisation. This directly complements the approach in which extrusion reduces the mycotoxin load, while subsequent pelleting determines the desired physical properties of the final product.

Jakovac-Strajn et al. [53] confirmed that the efficiency of mycotoxin reduction depends on toxin type and operational parameters (moisture content, screw speed, temperature, retention time). They highlighted that extrusion—especially at higher temperatures and appropriate moisture levels—can serve as a critical pre-processing step for materials destined for further pelleting, reducing both microbiological and toxicological risks.

Hoffmans et al. [44] comprehensively discussed process factors in cereal feed production (from harvest to final product), indicating that extrusion generally lowers mycotoxin content. The magnitude of this effect depends on temperature (often optimal for DON/OTA at 160–180 °C and for ZEN around 120 °C), moisture, and equipment configuration. The authors also noted that proper management of extrusion followed by pelleting helps maintain toxin levels “as low as reasonably achievable” for sensitive species.

Despite the clearly higher mycotoxin levels in feed mixtures containing 15% mould-infected cereal grains compared with those containing healthy grains, the obtained values remained within the permissible levels defined by current EU legislation [48]. DON, FB_1_, and AFB_1_ concentrations remained well below their regulatory limits, while OTA and T-2 approached, but did not exceed, their threshold values.

In practical feed applications, this indicates that even when lower-quality, microbiologically contaminated raw materials are used, the final feed product can still meet safety criteria and be safely used in animal nutrition without exceeding legal mycotoxin limits. It should also be noted that current regulations account for the presence of low background levels of mycotoxins, recognising their occurrence as unavoidable under typical crop production and storage conditions [54,55].

### 3.4. Microbiological Evaluation of Feed Mixtures

The obtained results showed that some samples of healthy cereal grains either did not contain mould colonies or contained only a few colonies, which could not be expressed as CFU. The remaining analysed samples contained microscopic fungi; however, their numbers were within the typical range for feed raw materials stored under proper storage conditions.

The highest total mould colony count was recorded for the mixture containing mould-infected maize (3B), reaching 1.70 × 10^5^ CFU·g^−1^ (Table 5). This may be attributed to its higher moisture content and the presence of a greater amount of simple carbohydrates, which promote fungal growth. The lowest colony count (4.80 × 10^4^ CFU·g^−1^) was observed in the mixture containing mould-infected barley (2B), which was characterised by lower moisture content and a harder grain structure, limiting the penetration and development of fungal spores.

In the other mixtures, the total mould counts were as follows: 7.23 × 10^4^ CFU·g^−1^ for 4B, 5.60 × 10^4^ CFU·g^−1^ for 5B, and 5.10 × 10^4^ CFU·g^−1^ for 1B. According to Kukier et al. [56], the microbiological contamination levels of compound feeds in Poland are typically below 10^5^ CFU·g^−1^ for fungi, below 10^2^ CFU·g^−1^ for Enterobacteriaceae, below 10^6^ CFU·g^−1^ for aerobic mesophilic bacteria, with *Clostridium* spp. titre not less than 0.001, and *C. perfringens* counts below 10^3^ CFU·g^−1^. In Germany, normal bacterial contamination levels range from 0.5 × 10^6^ to 1 × 10^7^ CFU·g^−1^, while mould contamination levels typically range from 5 × 10^3^ to 5 × 10^4^ CFU·g^−1^, depending on animal species and production group [57].

The pelleting process reduced mould colony counts in all analysed samples. The average reduction ranged from 27% (sorghum) to 65% (maize), confirming the effectiveness of thermo-mechanical treatment in lowering microbial contamination in feeds. The reduction in mould counts following pelleting is likely due to the combined effect of elevated temperature (70–90 °C) and pressure, which cause protein denaturation and damage to spore cell walls. Nevertheless, complete elimination of fungal microflora was not achieved in any sample, indicating that some spores may exhibit thermal resistance or remain dormant within the feed matrix.

The results indicate that pelleting represents an effective method for improving the microbiological quality of compound feeds; however, the efficiency of this process depends on raw material type, moisture content, chemical composition, and pelleting parameters. Therefore, regular microbiological monitoring of both raw materials and finished feeds should be an integral part of the quality assurance system (e.g., Hazard Analysis and Critical Control Points) in feed manufacturing facilities [58].

### 3.5. Statistical Analysis

**Figure 3:**
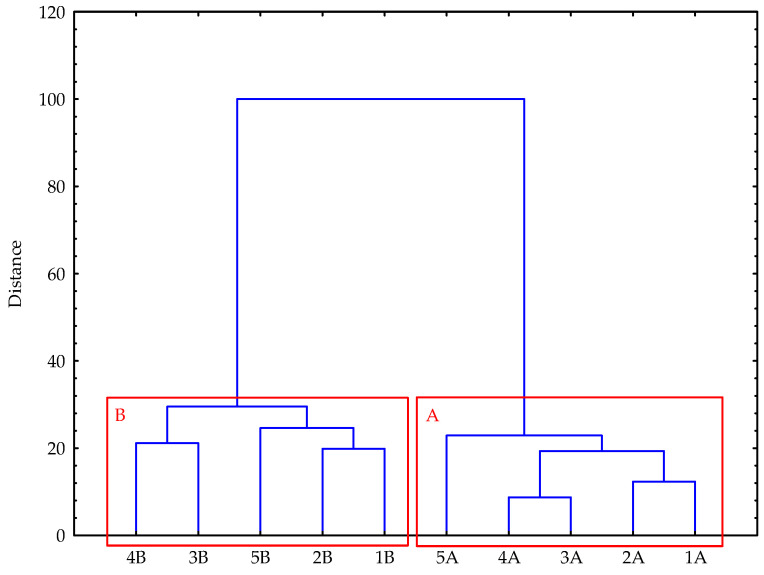
Dendrogram of the hierarchical cluster analysis showing two distinct groups: Cluster A—pellets produced from healthy cereal grains, and Cluster B—pellets produced from mould-infected grains. 1—pellet with wheat grain addition, 2—pellet with barley grain addition, 3—pellet with maize (corn) grain addition, 4—pellet with sorghum grain addition, 5—pellet with soybean grain addition, A—pellets produced from healthy cereal grains, B—pellets produced from mould-infected grains.

Based on the dendrogram (Figure 3), it can be concluded that, according to the rigorous Sneath criterion (33%), two distinct and relatively homogeneous groups (Clusters A and B) can be distinguished among the analyzed feed pellets. Cluster A comprises pellets produced from healthy cereal grains, which are generally characterized by lower physical and bulk density, reduced Holmen mechanical durability, higher pelletiser power demand, and lower mycotoxin content. This cluster also includes pellets with no detectable or only trace amounts of mould colonies (not quantifiable in CFU·g^−1^). In contrast, Cluster B consists of pellets produced from mould-infected grains, showing higher physical and bulk density, greater Holmen mechanical durability, lower pelletiser power demand, and elevated mycotoxin levels. Furthermore, the pellets in this cluster contained microscopic fungi, although their counts remained within the range typically observed in feed materials stored under proper conditions.

**Figure 4:**
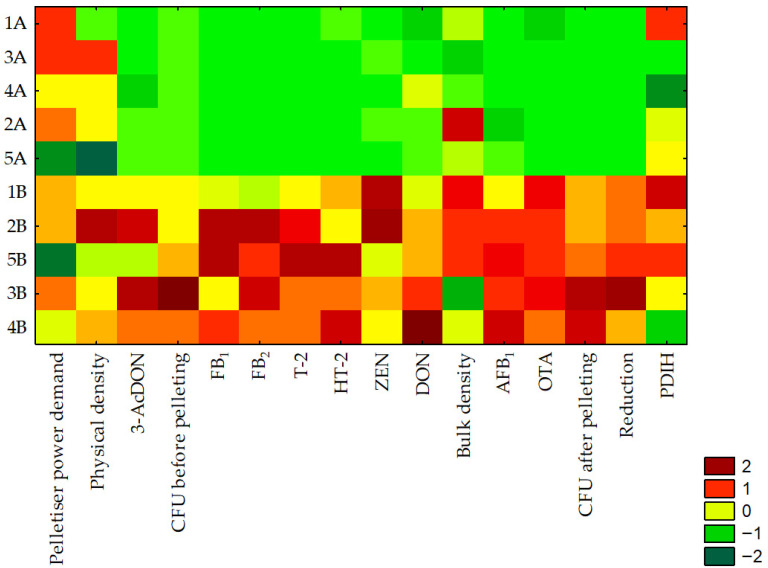
Heatmap with simultaneous clustering of pellets (from healthy and mould-infected cereal grains) and their physical and microbiological parameters. 1—pellet with wheat grain addition, 2—pellet with barley grain addition, 3—pellet with maize (corn) grain addition, 4—pellet with sorghum grain addition, 5—pellet with soybean grain addition, A—pellets produced from healthy cereal grains, B—pellets produced from mould-infected grains.

The analysis of the physical and microbiological properties of the examined pellets revealed two clearly distinct groups when both objects and features were simultaneously clustered (Figure 4). In the upper part of the similarity map, an area corresponding to pellets produced from healthy cereal grains (marked in green) was observed. Conversely, the lower part of the map represented pellets produced from mould-infected grains (marked in red). Among the pellets produced from healthy grains, those containing maize exhibited higher physical density values, whereas pellets containing barley showed the highest bulk density.

## 4. Conclusions

1. The use of mould-infected cereal grains at a 15% inclusion level as a feed component enabled the production of pellets with physical parameters comparable to those obtained from healthy raw materials. No significant deterioration was observed in key parameters such as physical density, bulk density, or Holmen mechanical durability.
2. The physical and bulk densities of the produced pellets remained within the acceptable range for compound feeds. Variants containing mould-infected grains (B) exhibited slightly lower density values; however, these differences were not large enough to negatively affect the functional quality of the feed.
3. The application of extrusion to mould-infected grains prior to pelleting increased the mechanical durability of the pellets by 4.02% compared with mixtures containing healthy grains. This result indicates that an appropriately selected thermo-mechanical processing regime can significantly enhance the mechanical properties of pellets, which is practically relevant for their transport and storage stability.
4. Mycotoxin levels in the final pellets increased following the incorporation of extruded mould-infected grains compared with healthy grains. However, all values remained within the permissible limits established by the European Commission. Particular attention should be given to ochratoxin A (OTA) and T-2 toxin, which approached their respective regulatory threshold levels.
5. The use of extrusion followed by pelleting represents an effective strategy for utilising mould-infected grains, reducing raw material losses and increasing the economic efficiency of feed production, provided that strict mycotoxin monitoring and control are maintained.
6. The extrusion–pelleting process effectively reduced mould colony counts, with an average reduction ranging from 27% (sorghum) to 65% (maize), confirming the high efficacy of thermo-mechanical treatment in reducing microbiological contamination. The mechanism involves exposure to elevated temperature and pressure, which damages fungal spore cell structures. However, complete elimination of fungal microflora was not achieved, indicating that some spores may exhibit thermal resistance or survive in a dormant state.

The results confirm the practical feasibility of controlled utilisation of microbiologically compromised raw materials while meeting the required safety and quality standards. Further research and development are essential to optimise extrusion and pelleting parameters, which could enhance mycotoxin reduction efficiency and support wider industrial implementation of this approach in the feed sector.

## Figures and Tables

**Table 1:** Composition of the pelletised mixtures.

Raw Material	Mixture Composition [%]									
	1A	1B	2A	2B	3A	3B	4A	4B	5A	5B
Meadow hay	40	40	40	40	40	40	40	40	40	40
Wheat bran	25	25	25	25	25	25	25	25	25	25
Fruit pomace	20	20	20	20	20	20	20	20	20	20
Wheat	15	-	-	-	-	-	-	-	-	-
Extruded wheat contaminated with mould spores	-	15	-	-	-	-	-	-	-	-
Barley	-	-	15	-	-	-	-	-	-	-
Extruded barley contaminated with mould spores	-	-	-	15	-	-	-	-	-	-
Maize	-	-	-	-	15	-	-	-	-	-
Extruded maize contaminated with mould spores	-	-	-	-	-	15	-	-	-	-
Sorghum	-	-	-	-	-	-	15	-	-	-
Extruded sorghum contaminated with mould spores	-	-	-	-	-	-	-	15	-	-
Soybean	-	-	-	-	-	-	-	-	15	-
Extruded soybean contaminated with mould spores	-	-	-	-	-	-	-	-	-	15

**Table 2:** Moisture content of raw and extruded feed materials.

Raw Material	Moisture ± SD [%]
Meadow hay	12.51 ± 0.19
Wheat bran	10.53 ± 0.15
Fruit pomace	9.35 ± 0.26
Wheat	11.65 ± 0.13
Barley	10.98 ± 0.11
Maize	12.76 ± 0.18
Soybean	11.47 ± 0.12
Sorghum	12.09 ± 0.21
Wheat contaminated with mould spores	19.98 ± 0.21
Barley contaminated with mould spores	20.11 ± 0.17
Maize contaminated with mould spores	22.58 ± 0.23
Soybean contaminated with mould spores	21.42 ± 0.20
Sorghum contaminated with mould spores	20.98 ± 0.25
Extruded wheat contaminated with mould spores	8.82 ± 0.08
Extruded barley contaminated with mould spores	8.16 ± 0.12
Extruded maize contaminated with mould spores	9.82 ± 0.08
Extruded soybean contaminated with mould spores	9.46 ± 0.10
Extruded sorghum contaminated with mould spores	9.28 ± 0.12

**Table 3:** Results of the study on the influence of incorporating extruded cereal grains infected with fungal spores into feed mixtures on the pelleting process (pelletiser power demand), mechanical durability, physical density, and bulk density of the produced pellets.

Pellet Type	Pelletiser Power Demand [kW]	Specific Energy Consumption [kWh·kg^−1^]	Physical Density [kg·m^−3^]	Bulk Density [kg·m^−3^]	Holmen Mechanical Durability (PDIH) [%]
1A	2.81± 0.11	0.0281	1131.17 ± 12.11	402.61 ± 5.21	79.11 ± 1.16
1B	2.76 ± 0.13	0.0276	1163.68 ± 18.25	410.82 ± 6.95	82.33 ± 0.99
2A	2.79 ± 0.18	0.0279	1159.40 ± 14.52	412.58 ± 5.38	73.51 ± 1.56
2B	2.74 ± 0.16	0.0274	1226.32 ± 9.83	409.27 ± 8.36	75.26 ± 1.18
3A	2.83 ± 0.10	0.0283	1199.34 ± 22.09	399.21 ± 6.59	67.24 ± 1.84
3B	2.78 ± 0.15	0.0278	1166.18 ± 25.83	397.47 ± 5.33	74.78 ± 1.53
4A	2.72 ± 0.14	0.0272	1159.22 ± 22.61	401.83 ± 6.86	63.15 ± 1.24
4B	2.70 ± 0.19	0.0270	1179.82 ± 29.48	404.02 ± 7.64	65.65 ± 1.49
5A	2.49 ± 0.18	0.0249	1063.63 ± 25.72	402.53 ± 6.13	74.74 ± 1.67
5B	2.45 ± 0.17	0.0245	1138.34 ± 24.35	409.89 ± 7.03	79.82 ± 1.26

1—pellet with wheat grain addition, 2—pellet with barley grain addition, 3—pellet with maize (corn) grain addition, 4—pellet with sorghum grain addition, 5—pellet with soybean grain addition, A—pellets produced from healthy cereal grains, B—pellets produced from mould-infected grains.

**Table 4:** Mycotoxin content in feed pellets.

Active Substance	Concentration [μg·kg^−1^_D.W._]									
	1A	1B	2A	2B	3A	3B	4A	4B	5A	5B
3-Acetyl-deoxynivalenol (3-AcDON)	3.11	18.50	8.39	36.18	3.83	39.81	1.19	24.89	8.21	11.80
Aflatoxin B_1_ (AFB_1_)	0.83	2.64	0.38	3.97	1.03	4.13	0.99	4.82	1.15	4.20
Deoxynivalenol (DON)	118.12	315.83	209.58	418.23	189.63	538.37	318.90	842.48	239.11	430.38
Fumonisin B_1_ (FB_1_)	11.37	68.11	12.45	258.11	9.38	112.65	7.52	187.52	11.89	265.12
Fumonisin B_2_ (FB_2_)	8.37	78.38	16.82	309.65	12.63	283.19	9.85	198.82	8.11	205.83
Ochratoxin A (OTA)	3.18	42.19	5.67	37.74	4.98	41.52	5.61	32.98	5.76	38.17
HT-2 toxin (HT-2)	7.42	37.00	2.63	29.73	4.63	38.17	3.52	61.50	4.87	67.10
T-2 toxin (T-2)	17.43	99.10	14.32	183.00	18.93	153.00	11.86	152.10	18.39	231.00
Zearalenone (ZEN)	2.13	17.30	3.62	18.95	2.54	9.83	1.19	7.20	1.82	6.50

1—pellet with wheat grain addition, 2—pellet with barley grain addition, 3—pellet with maize (corn) grain addition, 4—pellet with sorghum grain addition, 5—pellet with soybean grain addition, A—pellets produced from healthy cereal grains, B—pellets produced from mould-infected grains.

**Table 5:** Total number of mould colonies in the tested cereal grains and crop seeds before and after the pelleting process.

Pellet Type	Total Mould Colony Count Before Pelleting [CFU·g^−1^]	Total Mould Colony Count After Pelleting [CFU·g^−1^]	Reduction [%]
1B	5.10 × 10^4^	3.25 × 10^4^	36.28
2B	4.80 × 10^4^	3.27 × 10^4^	31.88
3B	1.70 × 10^5^	6.03 × 10^4^	64.53
4B	7.23 × 10^4^	5.27 × 10^4^	27.11
5B	5.60 × 10^4^	3.45 × 10^4^	38.39

1—pellet with wheat grain addition, 2—pellet with barley grain addition, 3—pellet with maize (corn) grain addition, 4—pellet with sorghum grain addition, 5—pellet with soybean grain addition, B—pellets produced from mould-infected grains.

## Data Availability

The original contributions presented in this study are included in the article. Further inquiries can be directed to the corresponding author.

## References

[1] Agriopoulou S., Stamatelopoulou E., Varzakas T. (2020). Advances in Occurrence, Importance, and Mycotoxin Control Strategies: Prevention and Detoxification in Foods. Foods.

[2] Streit E., Schatzmayr G., Tassis P., Tzika E., Marin D., Taranu I., Tabuc C., Nicolau A., Aprodu I., Puel O. (2012). Current Situation of Mycotoxin Contamination and Co-Occurrence in Animal Feed—Focus on Europe. Toxins.

[3] Gruber-Dorninger C., Jenkins T., Schatzmayr G. (2019). Global Mycotoxin Occurrence in Feed: A Ten-Year Survey. Toxins.

[4] Schaarschmidt S., Fauhl-Hassek C. (2018). The Fate of Mycotoxins During the Processing of Wheat for Human Consumption. Compr. Rev. Food Sci. Food Saf..

[5] Liu Y., Galani Yamdeu J.H., Gong Y.Y., Orfila C. (2020). A Review of Postharvest Approaches to Reduce Fungal and Mycotoxin Contamination of Foods. Compr. Rev. Food Sci. Food Saf..

[6] Soja J., Combrzyński M., Oniszczuk T., Gancarz M., Oniszczuk A. (2024). Extrusion-Cooking Aspects and Physical Characteristics of Snacks Pellets with Addition of Selected Plant Pomace. Appl. Sci..

[7] Boakye P.G., Okyere A.Y., Annor G.A. (2023). Impact of Extrusion Processing on the Nutritional and Physicochemical Properties of Intermediate Wheatgrass (*Thinopyrum intermedium*). Cereal Chem..

[8] Choton S., Gupta N., Bandral J.D., Anjum N., Choudary A. (2020). Extrusion Technology and Its Application in Food Processing: A Review. Pharma Innov. J..

[9] Szymczak P., Dziadowiec D., Andrzejewski J., Szostak M. (2023). The Efficiency Evaluation of the Reactive Extrusion Process for Polyethylene Terephthalate (PET). Monitoring of the Industrial Foil Manufacturing Process by In-Line Rheological Measurements. Appl. Sci..

[10] Kristiawan M., Della Valle G., Berzin F. (2022). Extrusion Simulation for the Design of Cereal and Legume Foods. Foods.

[11] Jain R., Goomer S. (2023). Understanding Extrusion Technology for Cereal–Pulse Blends: A Review. Cogent Food Agric..

[12] Riaz M.N. (2000). Extruders in Food Applications.

[13] Gulati P., Brahma S., Rose D.J., Ganjyal G.M. (2020). Chapter 13—Impacts of Extrusion Processing on Nutritional Components in Cereals and Legumes: Carbohydrates, Proteins, Lipids, Vitamins, and Minerals. Extrusion Cooking.

[14] Castells M., Marín S., Sanchis V., Ramos A.J. (2005). Fate of Mycotoxins in Cereals during Extrusion Cooking: A Review. Food Addit. Contam..

[15] Merrettig-Bruns U., Sayder B. Impact of Mycotoxins and Mouldy Maize Silage on the Biogas Process 2022. https://papers.ssrn.com/abstract=4084331.

[16] Kintl A., Vítěz T., Huňady I., Sobotková J., Hammerschmiedt T., Vítězová M., Brtnický M., Holátko J., Elbl J. (2023). Effect of Mycotoxins in Silage on Biogas Production. Bioengineering.

[17] Venslauskas K., Navickas K., Rubežius M., Žalys B., Gegeckas A. (2024). Processing of Agricultural Residues with a High Concentration of Structural Carbohydrates into Biogas Using Selective Biological Products. Sustainability.

[18] Cucina M., Tacconi C. (2022). Recovery of Energy and Nutrients from Mycotoxin-Contaminated Food Products through Biological Treatments in a Circular Economy Perspective: A Review. Agronomy.

[19] Wang J., Xing W., Huang X., Jin X., Yu H., Wang J., Song L., Zeng W., Hu Y. (2022). Smouldering of Storage Rice: Effect of Mouldy Degree and Moisture Content. Combust. Sci. Technol..

[20] (2015). Solid Biofuels—Determination of Moisture Content—Drying Method—Part 1: Total Mois-Ture—Reference Method.

[21] Ekstruzja soi i Zbóż|Presoil. http://presoil.pl/product-category/urzadzenia/tloczenie-soi-i-zboz/.

[22] Zasadzeń M. (2018). Analiza niezawodności wybranych maszyn w przedsiębiorstwie produkcyjnym—Studium przypadku. Cross-Border Exchange of Experience Production Engineering Using Principles of Mathematics: Modern Mathematical Methods in Engineering 3mi, 22.1.–24.1. 2018, Horni Lomna.

[23] Cwalina P., Obidziński S., Sienkiewicz A., Kowczyk-Sadowy M., Piekut J., Bagińska E., Mazur J. (2025). Production and Quality Assessment of Fertilizer Pellets from Compost with Sewage Sludge Ash (SSA) Addition. Materials.

[24] (2016). Solid Biofuels—Determination of Bulk Density.

[25] (2016). Solid Biofuels—Determination of Mechanical Durability of Pellets and Briquettes.

[26] (2012). Ziarno Zbóż i Przetwory Zbożowe—Pobieranie Próbek.

[27] Technologia Produkcji Pelletu—TMB Polska. https://tmbpolska.pl/produkcji-pelletu-technologia/.

[28] Teixeira Netto M.V., Massuquetto A., Krabbe E.L., Surek D., Oliveira S.G., Maiorka A. (2019). Effect of Conditioning Temperature on Pellet Quality, Diet Digestibility, and Broiler Performance. J. Appl. Poult. Res..

[29] Bastiaansen T.M.M., de Vries S., Martens B.M.J., Benders R.T., Vissers E., Dijksman J.A., Hendriks W.H., Thomas M., Bosch G. (2024). Identifying Feed Characteristics That Affect the Pellet Manufacturing of Livestock Diets Containing Different Coproducts. Clean. Circ. Bioecon..

[30] Schroeder B., Andretta I., Kipper M., Franceschi C.H., Remus A. (2020). Empirical Modelling the Quality of Pelleted Feed for Broilers and Pigs. Anim. Feed Sci. Technol..

[31] Çitil E., Marakoğlu T. (2024). Effect of Feed Materials on Pellet Properties, Capacity and Energy Consumptions Values. J. Agric. Fac. Gaziosmanpasa Univ..

[32] Feed Safety|FEFAC. https://fefac.eu/priorities/feed-safety/.

[33] Peng F., Xiang R., Fang F., Liu D. (2022). Analysis of Feed Pelleting Characteristics Based on a Single Pellet Press Device. Int. J. Agric. Biol. Eng..

[34] Ungureanu N., Vladut V., Voicu G., Dinca M.-N., Zabava B.-S. (2018). Influence of Biomass Moisture Content on Pellet Properties—Review. Eng. Rural Dev..

[35] Rawski M., Mazurkiewicz J., Kierończyk B., Józefiak D. (2020). Black Soldier Fly Full-Fat Larvae Meal as an Alternative to Fish Meal and Fish Oil in Siberian Sturgeon Nutrition: The Effects on Physical Properties of the Feed, Animal Growth Performance, and Feed Acceptance and Utilization. Animals.

[36] Rosani U., Ayuningsih B., Susilawati I., Hernaman I., Indriani N.P., Putri G.N.R., Imanda A.R., Begna R. (2025). Physical and Nutritional Characteristics of Indigofera, Gamal, and Cassava-Based Pellets for Sustainable Livestock Feed. Adv. Anim. Vet. Sci..

[37] Oliveira L.C., Schmiele M., Steel C.J. (2017). Development of Whole Grain Wheat Flour Extruded Cereal and Process Impacts on Color, Expansion, and Dry and Bowl-Life Texture. LWT.

[38] Combrzyński M., Wójtowicz A., Mitrus M., Oniszczuk T., Matwijczuk A., Pawelczyk P., Mościcki L. (2019). Effect of Starch Type and Screw Speed on Mechanical Properties of Extrusion-Cooked Starch-Based Foams. Int. Agrophys..

[39] Massuquetto A., Durau J.F., Schramm V.G., Netto M.T., Krabbe E.L., Maiorka A. (2018). Influence of Feed Form and Conditioning Time on Pellet Quality, Performance and Ileal Nutrient Digestibility in Broilers. J. Appl. Poult. Res..

[40] Mohammadi Ghasem Abadi M.H., Moravej H., Shivazad M., Karimi Torshizi M.A., Kim W.K. (2019). Effect of Different Types and Levels of Fat Addition and Pellet Binders on Physical Pellet Quality of Broiler Feeds. Poult. Sci..

[41] Angulo E., Brufau J., Esteve-Garcia E. (1996). Effect of a Sepiolite Product on Pellet Durability in Pig Diets Differing in Particle Size and in Broiler Starter and Finisher Diets. Anim. Feed Sci. Technol..

[42] Amerah A.M., Ravindran V., Lentle R.G., Thomas D.G. (2008). Influence of Feed Particle Size on the Performance, Energy Utilization, Digestive Tract Development, and Digesta Parameters of Broiler Starters Fed Wheat- and Corn-Based Diets. Poult. Sci..

[43] Chewning C.G., Stark C.R., Brake J. (2012). Effects of Particle Size and Feed Form on Broiler Performance. J. Appl. Poult. Res..

[44] Hoffmans Y., Schaarschmidt S., Fauhl-Hassek C., van der Fels-Klerx H.J. (2022). Factors during Production of Cereal-Derived Feed That Influence Mycotoxin Contents. Toxins.

[45] Janić Hajnal E., Babič J., Pezo L., Banjac V., Filipčev B., Miljanić J., Kos J., Jakovac-Strajn B. (2024). Reduction of Alternaria Toxins via the Extrusion Processing of Whole-Grain Red Sorghum Flour. Foods.

[46] Pinotti L., Ottoboni M., Giromini C., Dell’Orto V., Cheli F. (2016). Mycotoxin Contamination in the EU Feed Supply Chain: A Focus on Cereal Byproducts. Toxins.

[47] Scarpino V., Bresciani A., Blandino M. (2024). The Effects of the Extrusion Process Used for the Production of Maize Snacks and Pasta on the Free, Bound, and Total B Fumonisin Contents. LWT.

[48] (2024). Rozporządzenie Komisji (UE) 2024/1038 z Dnia 9 Kwietnia 2024 r. Zmieniające Rozporządzenie (UE) 2023/915 w Odniesieniu do Najwyższych Dopuszczalnych Poziomów Toksyn T-2 i HT-2 w Żywności. https://eur-lex.europa.eu/legal-content/PL/ALL/?uri=CELEX:32024R1038.

[49] Chełkowski J. Mikotoksyny, Grzyby Toksynotwórcze i Mikotoksykozy. http://www.cropnet.pl/dbases/mycotoxins.pdf.

[50] Schrenk D., Bignami M., Bodin L., Chipman J.K., del Mazo J., Grasl-Kraupp B., Hogstrand C., Hoogenboom L., Leblanc J., EFSA Panel on Contaminants in the Food Chain (CONTAM) (2020). Risk Assessment of Aflatoxins in Food. EFSA J..

[51] Janić Hajnal E., Babič J., Pezo L., Banjac V., Čolović R., Kos J., Krulj J., Pavšič-Vrtač K., Jakovac-Strajn B. (2022). Effects of Extrusion Process on *Fusarium* and *Alternaria* Mycotoxins in Whole Grain Triticale Flour. LWT.

[52] Khan G.Q., Prestløkken E., Lund P., Hellwing A.L.F., Larsen M. (2023). Effects of the Density of Extruded Pellets on Starch Digestion Kinetics, Rumen Fermentation, Fiber Digestibility and Enteric Methane Production in Dairy Cows. J. Anim. Physiol. Anim. Nutr..

[53] Jakovac-Strajn B., Babič J., Pezo L., Banjac V., Čolović R., Kos J., Miljanić J., Janić Hajnal E. (2025). Mitigation of Mycotoxin Content by a Single-Screw Extruder in Triticale (x *Triticosecale wittmack*). Foods.

[54] Torres A.M., Palacios S.A., Yerkovich N., Palazzini J.M., Battilani P., Leslie J.F., Logrieco A.F., Chulze S.N. (2019). Fusarium Head Blight and Mycotoxins in Wheat: Prevention and Control Strategies across the Food Chain. World Mycotoxin J..

[55] Palumbo R., Crisci A., Venâncio A., Cortiñas Abrahantes J., Dorne J.-L., Battilani P., Toscano P. (2020). Occurrence and Co-Occurrence of Mycotoxins in Cereal-Based Feed and Food. Microorganisms.

[56] Kukier E., Kwiatek K., Goldsztejn M., Grenda T. (2014). Jakość Mikrobiologiczna Pasz. Pasze Przem..

[57] LLFG Sachsen-Anhalt (2011). Orientierungswerte zur Beurteilung der Mikrobiologischen Qualität von Futtermitteln (nach VDLUFA Methodenbuch III 28.1.4, 8. Ergänzung 2011).

[58] Goldsztejn M., Grenda T., Kozak B., Kozieł N., Kwiatek K. (2022). Microbiological Contamination of Feed-Current Hazards and New Challenges. Med. Weter..

